# Perspectives of men on antenatal and delivery care service utilisation in rural western Kenya: a qualitative study

**DOI:** 10.1186/1471-2393-13-134

**Published:** 2013-06-21

**Authors:** Titus K Kwambai, Stephanie Dellicour, Meghna Desai, Charles A Ameh, Bobbie Person, Florence Achieng, Linda Mason, Kayla F Laserson, Feiko O ter Kuile

**Affiliations:** 1 KEMRI/CDC Research and Public Health Collaboration, Kisumu, Kenya; 2 Ministry of Public Health and Sanitation, Nairobi, Kenya; 3 Liverpool School of Tropical Medicine, Liverpool, UK; 4 Division of Parasitic Diseases and Malaria, Center for Global Health, Centre for Diseases Control and Prevention, Atlanta, GA, USA; 5 National Centre for Emerging and Zoonotic Infectious Diseases, Centres for Disease Control and Prevention, Atlanta, GA, USA; 6 Department of Public Health and Policy, University of Liverpool, Liverpool, UK; 7 Center for Global Health, Centres for Disease Control and Prevention, Atlanta, GA, USA

**Keywords:** Pregnancy, Antenatal care, Delivery care, Decision making, Male involvement

## Abstract

**Background:**

Poor utilisation of facility-based antenatal and delivery care services in Kenya hampers reduction of maternal mortality. Studies suggest that the participation of men in antenatal and delivery care is associated with better health care seeking behaviour, yet many reproductive health programs do not facilitate their involvement. This qualitative study conducted in rural Western Kenya, explored men’s perceptions of antenatal and delivery care services and identified factors that facilitated or constrained their involvement.

**Methods:**

Eight focus group discussions were conducted with 68 married men between 20-65 years of age in May 2011. Participants were of the Luo ethnic group residing in Asembo, western Kenya. The area has a high HIV-prevalence and polygamy is common. A topic guide was used to guide the discussions and a thematic framework approach for data analysis.

**Results:**

Overall, men were positive in their views of antenatal and delivery care, as decision makers they often encouraged, some even ‘forced’, their wives to attend for antenatal or delivery care. Many reasons why it was beneficial to accompany their wives were provided, yet few did this in practice unless there was a clinical complication. The three main barriers relating to cultural norms identified were: 1) pregnancy support was considered a female role; and the male role that of provider; 2) negative health care worker attitudes towards men’s participation, and 3) couple unfriendly antenatal and delivery unit infrastructure.

**Conclusion:**

Although men reported to facilitate their wives’ utilisation of antenatal and delivery care services, this does not translate to practice as adherence to antenatal-care schedules and facility based delivery is generally poor. Equally, reasons proffered why they should accompany their wives are not carried through into practice, with barriers outweighing facilitators. Recommendations to improve men involvement and potentially increase services utilisation include awareness campaigns targeting men, exploring promotion of joint HIV testing and counselling, staff training, and design of couple friendly antenatal and delivery units.

## Background

Most cultures, especially in Africa, regard pregnancy and delivery as a female domain; therefore, men are often not expected to accompany their wives to the antenatal care (ANC) clinic or be present during delivery [1,2]. As decision maker for the family, decisions around when, where and even if, a woman should have access to healthcare often fall to men. Particularly in patriarchal societies, the health status of women and children suffer especially where women have little control over family finances, little say in decision making and restricted freedom of movement [3]. A study in Tanzania found that households headed by men were associated with more home based deliveries [4], while in Pakistan high decision making power by men was linked to low utilisation of ANC and delivery care services [5]. The International Conference on Population and Development in Cairo in 1994 [6] and the Fourth World Conference on Women, Beijing 1995 [7] pointed towards the need for involving and encouraging men to take responsibility for their sexual and reproductive behaviour, advocating that men are in a position to change attitudes and practice through their positions as community, religious and political leaders. However, they should also take individual responsibility as husbands and fathers to become involved in changing social attitudes including taking responsibility for reproductive health issues.

However, their involvement has been slow, and the lack of progress is a likely contributor to the sub-optimal advancement towards the achievement of the United Nations Millennium Development Goal (MDG) 5: reduce maternal mortality by 75% between 1990 and 2015 [8]. Attempts to encourage men to attend for ANC and childbirth have been promoted by individual health facilities in Kenya, with mixed successes and failures similar to reports from other resource poor settings [9]. Most efforts have been centered around sexual and reproductive health issues such as condom use, family planning decision making and prevention of mother to child transmission (PMTCT) of HIV [10-12]. Figures are not available on the number of male partners that accompany their wives, either for ANC or delivery care, but based on anecdotal evidence these are low in the Kenyan setting. In order to improve men’s participation, reasons for their poor or reluctant involvement need to be explored. Yet, to date, with the exception of a study around HIV testing and counselling in ANC set up in Nairobi, the largest city in Kenya with a population of around 3 million inhabitants [13], men’s perceptions have not been explored in the Kenyan setting and information from other resource poor settings is limited [2,9,14-16] and may not be transferrable to the current setting due to unique cultural and economic factors.

This study was designed to provide insight into men’s perceptions of maternal health care services and to identify factors that facilitate or constrain men’s involvement in ANC and delivery care in western Kenya. The specific research questions we sought answers for were: what were men’s perceptions of their role during their wives pregnancy? What did they understand about ANC and delivery care services? What factors prevented them from accompanying their wives to ANC and delivery care services? What factors would enable them to accompany their wives for ANC or delivery care?

## Methodology

### Study area

This study was carried out in Asembo, a rural district in Nyanza Province, western Kenya. Fishing and subsistence farming are the main economic activities in the area [17]. This is a culturally homogeneous population with the majority of the residents belonging to the Luo ethnic group. Polygamy is practiced by many communities in Kenya; Polygyny is highest in Nyanza Province, and about 21% of women are in a polygamous relationship [18].

The health indicators and utilisation of health facilities are poor in the study area compared to the rest of Nyanza Province and Kenya. The maternal mortality ratio (MMR) in the area was estimated to be 606 per 100,000 live births in 2008, compared to 488 nationally [18]. The proportion of home based deliveries were 83% and only 36% of pregnant women made the recommended four ANC visits, compared to national figures of 55% and 47% respectively [18,19]. Like the rest of Nyanza Province, the area has a higher HIV-prevalence of 14.9% compared to 7.1% for the rest of Kenya [20]. In this context, ANC is the care provided by a skilled attendant during pregnancy. Facility-based delivery care refers to birth attendance by a skilled birth attendant in a health facility. ANC is provided at the ‘mother and child clinic (MCH)’, most of which have limited space and may have more than one health care worker (HCW) offering ANC, postnatal and immunization services in the same room. Labour wards in most facilities do not have barriers or separate rooms for every mother. Within the study area there are four Government owned dispensaries, a health centre and one mission hospital all offering ANC and delivery care services. Only the Mission hospital has an ambulance. Due to a shortage of HCWs, dispensaries are open only during the day and may not be open at the weekends.

## Methods

A qualitative approach was adopted for this study as little is currently known about the views of men towards ANC and delivery care services in this setting. The study thus aimed to explore those issues that the men themselves felt to be important. The focus group discussions (FGD’s) were conducted during May 2011 with participants recruited from 33 villages around Lwak Mission Hospital which are part of an enhanced population-based morbidity surveillance system conducted by the Kenya Medical Research Institute/Centers for Disease Control (KEMRI/CDC) in this area [21]. Purposive sampling was used to ensure a homogenous make-up of each FGD along similar age group and distance from the health facility, in order to better understand any potential differences among older and younger men, and any influence of access to care. The focus groups were stratified by age category: 20-40 years (n=4, 34 total participants); 41-65 years (n=4, 34 total participants) and by distance to the central health facility (within 2.5 km or further; 2 in each group). Recruitment was assisted by village reporters (VRs) who act as community liaisons for KEMRI/CDC studies. A list of characteristics of men for each of the 8 FGDs were provided to the VR who referred individuals from their respective villages. Inclusion criteria stipulated participants had to be native Luo speakers and married to a woman who was pregnant, or who had been pregnant within the last 5 years.

Eight FGDs comprising 8 to 10 participants were conducted. All took place at a central location in Lwak during the afternoon hours when participants were available. The FGDs were conducted in Dholuo by a trained Luo moderator with support from the PI working as note taker. A topic guide was used to provide direction for the discussion but allowed deviations to pursue any emerging avenue of enquiry in further detail [22,23]. The topics covered a) knowledge of ANC and delivery care, b) perceptions of the male role in influencing utilisation of care c) men’s roles and participation in ANC and delivery care, and d) potential barriers to their participation in care.

The FGD proceedings were audio recorded using a digital voice recorder, transcribed and translated into English language by two experienced research assistants, fluent in both Dholuo and English. The “Thematic framework approach” was used to analyse the data [22]. This process allowed us to use a mixture of deductive and inductive analysis [24]. The analysis was primarily deductive, searching for information to answer the research questions using pre-set coding. However, it also became inductive as new themes emerged from the data. The steps taken were: the FGD transcripts were imported into QSR NVivo 9 software (QSR International Pty Ltd). Reading and re-reading of the transcripts was undertaken to allow familiarisation with the text, brief notes were made to document the major issues emerging. This was followed by a line by line, micro analysis using open coding. The codes were then assembled into potential themes and a thematic chart was then developed in MS Word. The themes were compared across the transcripts and specifically the different groups, to establish the range and similarities of the participants’ perceptions, experiences and views. They were also checked to ascertain that they had not been over or under represented. The next step involved axial coding – re-contextualising the original codes along with the text. Narrative text was applied around the themes, with quotes used to illustrate the text and communicate its meaning to the reader. TKK coded the transcript and all codes and emerging themes were reviewed by LM.

### Ethical approval

Ethical approval was obtained from the Liverpool School of Tropical Medicine (LSTM), The Centers for Disease Control and Prevention (Atlanta, Georgia, USA) and The Kenya Medical Research Institute Ethical Review Boards. Verbal informed consent for participation and audio recording of the discussions was obtained from each participant. Password protection of soft data and use of key and lock for hard copy data was employed to guarantee confidentiality.

## Results

From the analysis of data, 7 major themes were evident (female roles, male roles, healthcare worker attitudes, benefits of facility-based maternity care, benefits of men’s attendance, barriers to men’s attendance, logistics and the role of Traditional Birth Attendants (TBAs)). The subthemes of male dominance, negativity and distrust between spouses, also emerged within the main themes.

### Pregnancy support as a female role

Many men reported that primary pregnancy support was most often provided by other women. Overall, men told us that males were often excluded from a supporting role, even as part of a couple. Many mentioned that disclosure of pregnancy was rarely made to them in the first instance, despite some men’s wish to be the first to know. Most men reported that women typically chose to tell their mothers in law, co-wives, friends or trusted female neighbours before their husbands. Some men told us that they thought delayed or even non-disclosure to their husband was often due to women’s ‘shyness’ in discussing ‘female issues’ or fear of the husbands’ reaction to an unplanned pregnancy (which many were).

***“****she did not tell me but I saw her stomach growing, is when I realised that she is pregnant so when I asked her slowly that what’s happening nowadays that you’re gaining weight, that is when she told me”****(FG7, P3)***.

*“She told one of her in- laws about the pregnancy, therefore that is how I got the information. She was scared to tell me because the family planning method failed, so she thought her in law was going to convince me not to beat her up”****(FGD 1,P7)***.

### Men as the provider

Men typically reported their role during pregnancy as providers. Many participants believed that a woman should take rest and refrain from too much manual labour during pregnancy and so men took on some of her domestic duties e.g. fetching water, splitting firewood and cultivating land, activities thought to be detrimental to the health of both the mother and her unborn child. Yet, despite this belief, a few men reported assisting their wives only late in their pregnancy.

*“for the mother, tough work will give her and the child problems, so you give her light work so that she can have a good life… bringing flour and fetching fire wood are some of the jobs that we help them [with] when they are pregnant” (****FGD 2, P8)***.

Some men also reported purchasing additional food for their wife to supplement her diet, which was considered important for her health and her unborn child. A few men told us that tight fitting clothes could affect the health of the foetus; therefore they bought maternity clothes for their pregnant spouses. Just a few older participants mentioned reminding their wife about clinic appointments and occasionally accompanying them to clinic, but only if they were ill or there was a perceived complication of the pregnancy. Emotional support was mentioned, by some participants in both age groups, but only when the moderator specifically asked about it.

### Men as decision maker

The patriarchal role of Luo men was also reflected in their identification of being in charge of household money and thus the decision maker of matters pertaining to maternity care. Few men reported discussing such matters with their wives, and was another example of the male dominant role which emerged during the discussions.

*“I think you are the one to choose because you are the husband [and] you are the owner of the money, so when you are looking for a health facility you are the one to choose according to your money…”****(FGD 2, P1)***.

*“I will tell her –‘if you refuse to do as I say, go away’…and you know if you tell your wife to go away, tears will roll down, so it will force her to do as I say”****(FGD1, P3)***.

As well as for financial reasons, some men also justified making decisions based on their wives poor health seeking behaviour, due to ignorance and/or laziness. As a result some men occasionally had to ‘*force’* their wives to seek care from facility-based services or sometimes, the TBA.

“*Even if the clinic is near, you have to force her to go to the clinic. So with laziness they will not make it even if it is free of charge”****(FGD 5, P2)***.

### Men’s perceived benefits of health facility ANC and delivery care

Knowledge about the services provided during ANC care included confirmation of pregnancy, detection of complications and testing for HIV. Some men also mentioned “*checking the position of the baby in the stomach*”. Weight monitoring, malaria tests, blood pressure measurements, temperature and blood group typing were also mentioned by a minority of participants. Most were aware that medication could be issued, but they could neither name the drugs nor their uses. Irrespective of the depth of knowledge of the services provided, there was strong opinion voiced across all FGDs that wives should attend ANC. However, the TBA was also mentioned, though less often, as a source of healthcare during pregnancy – particularly a source of advice because of their experience.

*“When she was pregnant I told her to go to clinic or I can tell her to go to the nearest traditional birth attendant who can give her advice on how to take care of herself and the child she is carrying” ***(FGD 7, P1)**.

Most men reported that a hospital or health facility was the best place to deliver a baby. The hospital was seen as hygienic and would “*prevent infection by any disease like tetanus.”* Complications could be better managed because they were staffed by qualified personnel and had the necessary equipment. Hospitals could handle birth complications such as delivery of a big baby, stitching ‘*cuts*’. Other merits for hospital based deliveries were prevention of HIV transmission from mother to child, and vaccination after delivery.

*“At first that is where [hospital] there is a specialized doctor that if you are found having a problem, then he knows what he can do. Secondly that is where there are equipments that can be used on that person in case of any problem…there is also good care and observation” (****FGD 2, P4)***.

*“…there are women who can get pregnant and they are HIV positive. If they go for delivery in a health facility and it is discovered, they can find a way to help the child not to get infected with HIV…” (****FGD 6, P1)***.

### Logistics and the TBA

However, despite this stated preference of hospital based delivery care a few men reported that using a TBA was sometimes inevitable because whilst delivery care services were affordable in hospital, the flexibility of payment for TBA services made payment easier. TBA charges were negotiable and could be paid in kind or by instalments.

*“…she can discuss with the TBA and come to an agreement. Sometimes you will pay her later if you still don’t have money… but you know in hospital when you are being discharged you have to pay the entire amount”****(FGD 6, P5)***.

*“There is no [fixed] rate of payment for TBA services; it depends on how you talk to her…. now the day her husband is coming to take her is when he can give something small as gratitude”****(FGD1, P1)***.

Furthermore, the TBA was viewed by men as local, making access much easier, particularly when transport was not available. Many men mentioned that the health facilities are far and roads are impassable, especially, during the rainy seasons. Other reasons men shared for utilising the TBA appeared to stem from the wives preference and perceived ‘manipulation’ of the situation i.e. they deliberately failed to plan for the delivery, were ignorant of the expected date of delivery, and intentionally left it too late to go to hospital once labour had started, thus the TBA was the only option left. The quotes below also serve to illustrate the subtheme of distrust which commonly emerged from the discussions.

*“…most women buy time until they are not able to control [pain] so they just go to the TBA”****(FGD 7, P3)***.

*“…there are some who forget their delivery dates and when labour comes they just deliver in their houses or TBA’s…there are some who do that and when you ask…, she tells you that it just came suddenly…”****(FGD 8, P1)***.

### Benefits of male attendance to ANC

Interestingly, most participants across all FGDs reported that it was beneficial to accompany their wives for ANC, to get first-hand information about the health of their wives and unborn babies. Some men believed that their wives either distorted or withheld some information after clinic visits; the implication being that this was sometimes a deliberate act. A lack of trust meant they needed to hear it directly from the HCW, and was again another example of the distrust between the men and their wives. A few participants, especially the younger men, also reported they would be able to remind their spouses to follow the doctors’ instructions.

*“For me, the benefit that I will get…[is] first-hand information, that is, what she is being told I also hear, not that I wait for a report that can be distorted, she may not tell me others” (****FGD 1,P5)***.

Accompanying a wife to ANC was reported by a few younger men as a way to discover her HIV status, whilst others felt it encouraged disclosure between couples. Receiving counselling on HIV/AIDS and prophylaxis to prevent transmission of HIV to the unborn child was also mentioned as an important reason to visit the clinic by some men in both age groups. However, HIV testing was also perceived as a barrier for ‘*other*’ men and just occasionally, as a reason for their preventing wives from attending clinic.

*“… if we go with her to the clinic, we can be tested and get out of there knowing our status [of HIV]… if one person is positive and another negative then we can be told how to stay together”****(FGD 1, P4)***.

*“He is an old man but he doesn’t want his wife to go to clinic because, he says that if she goes then his status (HIV) will be discovered..... He knows that he is infected but he didn’t tell the wife this thing”****(FGD 3, P7)***.

### Benefits of male attendance to delivery care

As with ANC care, participants also identified reasons they should accompany their wife during the birth. They could organise transport if referral is required, be available to sign consent for surgery, and make decisions if complications occurred. Some men reported that their wives, in some cases, would or could not, make major decisions in their absence, another example of the dominant male role in the partnership.

*“When you are with her it shows contact, you are aware of what is going on there and in case of any complications or even if any referral might be needed, you may decide on what you can do…” (****FGD 1, P6)***.

A few participants pointed out that they would be able to pay for any necessities, and importantly, could also prevent their baby from being exchanged for another.

*“… nowadays the world has changed, someone can switch your child… there is a possibility that if someone wanted a baby boy and gives birth to a baby girl, they may exchange for your baby boy”****(FGD 7, P1)***.

Some men reported that their role was to provide love and encouragement during labour.

*“…being there [during delivery] gives her courage, so even if she is now pushing the child, she pushes it with happiness since you are there [laughter]…”****(FGD 1, P7)***.

Despite the perceived benefits of accompanying their wives, very few men mentioned that they did this in practice. Indeed they only spoke of going with their wives when there were specific health issues, pregnancy complications, or laboratory tests were necessary. As such this seemed to be a ‘one off’ act not leading to routine accompaniment.

### Barriers to attendance at ANC and delivery care

Most participants across the FGDs echoed three issues preventing them from accompanying their wives for either ANC or delivery care. Firstly this was seen as a ‘female’ role and thus the responsibility of mother-in-laws or co-wives rather than the male.

Secondly, as head of household and provider, men’s focus was on economic activity which was more important for them to concentrate on at this time. Interestingly, a couple of participants admitted that they had *pretended* to be busy so as to avoid accompanying the spouse for ANC. In this vein, some participants reported that they would readily accompany their wives to ANC if they would be given priority in the queue before women who were unaccompanied (by a male partner). This is practiced in some clinics in a neighbouring district, but it is not a Government policy.

*“… if you go with your wife, they [should] consider those who have come with their husbands [and] give them the first priority…”****(FGD7, P5)***.

The third barrier stemmed from the negative attitude that HCWs had towards men participating in ANC or delivery care. Most men in all FGDs reported that they had, or had heard anecdotally that other men had been ignored by HCWs, subjected to unfriendly attitude or even abusive language.

*“ I have heard others say that if they go to the clinic, the sisters there are harsh to them so they fear going to be harassed because it leaves them in a state that they don’t like’****(FG7, P1)***.

Most men who had accompanied their wives for delivery similarly reported that maternity staff did not allow them to enter the delivery rooms; therefore, they choose to stay away feeling that they were not wanted there.

*“… those people [HCW] are so harsh and they don’t want a man to step inside [delivery room]… there is nothing you can do because you cannot see her although you wish to be near her, so it is even better for you to stay away and do some other things that can help her after delivery”****(FGD 8, P7)***.

However, there were exceptions where few men were allowed into the labour ward but chose not to, either because they thought their wife would not like to be seen in labour, or they were afraid of what they would see.

*“But when I entered this room I found some women beating walls with their hands and I felt afraid that I am also going to see my wife doing that. I went and stood outside” (****FG7 P4)***.

There were mixed responses when men were asked about witnessing the birth of their babies. Some older men were more likely than the younger men to feel that it was culturally disrespectful for a man to see a woman giving birth.

*“seeing the way the child is coming out and that is the same place that you would like to have sex at…it can’t be good because you may lose the urge”****(FGD 4, P8)***.

Interestingly, whilst acknowledging that it was not true, some participants frequently reported that traditionally, Luo people believed that the presence of the husband at the time of delivery delays the birth of the baby.

## Discussion

This study is one of the few which examine men’s perceptions of facility-based ANC and delivery care in Africa, and of perceived factors that encourage or hinder them from accompanying their wives to the health facility. The influence of men on women’s sexual and reproductive health outcomes have been increasingly recognised since the International Conference on Population and Development in Cairo in 1994 [6]. Better pregnancy outcomes have been shown when men are directly involved in maternal health care through ANC attendance and pregnancy support [25,26]. However, with poor rates of both ANC and hospital based delivery care it is important to find ways of improving the current situation.

Many of our findings echo the limited evidence from other qualitative studies carried out from the male perspective, and the more numerous studies both qualitative and quantitative focusing on the female. The main barriers identified were: pregnancy support is a female role, the male role is one of provider and decision maker, HCW’s have a negative attitude towards men being involved in maternity care.

There were two positive findings that could be utilised in future attempts to involve men in ANC and delivery care. Evidence from our FGD’s showed that most men in both age groups were strongly in favour of their wives having ANC care and a hospital delivery, and felt that it could be beneficial to accompany them, despite rarely doing this in practice.

### Men’s rationale for accompanying their wife for ANC and delivery

Participants put forward many reasons to accompany wives to ANC clinic, yet not all reasons were positive. The narratives of many men had an undercurrent of distrust e.g. wives would fail to disclose important information to them, or distort the truth – seemingly in some instances on purpose. Alternatively they would fail to understand or remember important information accurately. As a consequence some men told us that they could not rely on their wives to seek the best healthcare for themselves. A study in South Africa similarly reported that women did not tell their husbands what had occurred at the clinic, however, there was no sense of distrust conveyed in this context [24]. A few men when talking about distrust characterized their wives as deliberately stalling during their labour so that the TBA was the only recourse for delivery.

On a more positive note, some men felt that promotion of voluntary counselling and testing for HIV and their involvement in PMTCT was a reason for going to the clinic, a finding consistent with the conclusions of a Ugandan study [27]. However, this requires further studies in this specific community in western Kenya, particularly as pregnant women reported the HIV test as a reason for avoiding ANC care in a study conducted in a nearby district [28]. Few men also reported that weight monitoring, provision of bed nets to prevent malaria, blood pressure measurements, temperature and blood group typing were reasons for seeking care.

In practice, men were more likely to accompany their spouses to hospital if there was a perceived complication, consistent with findings of Odimegwu et al from Nigeria [29]. It is possible that this could also be the only time their wives use facility-based ANC or delivery care. Therefore, it is important, during the initial ANC attendance and any community health education, to ensure that the populations understand that facility care is to be utilised, not just by those with complications, a misconception reported in other studies conducted in developing countries [30-32].

### Men’s perceived role during pregnancy and delivery

The study was undertaken in a patriarchal society and this was clear in the dominant role articulated by the participants. Most men reported that their role in the pregnancy was typically as provider and decision maker – providing practical help when their wives could not undertake the arduous domestic tasks allotted to them, a finding consistent with other research [29]. Men saw themselves as dominant in decision making. Whilst occasionally some admitted that they would discuss issues with their wives, more often men took a unilateral decision, with some reporting they would ‘force’ their wife if she did not agree with their decisions (although this was not necessarily physical force). It is noteworthy that if the men were the main decision makers, and as proactive in ‘forcing’ their wives to attend for health facility care as they stated, then we could perhaps expect a higher female utilisation rate than has occurred to date. Unlike our study, a previous quantitative study in the same setting reported that majority of the women (87%) made these decisions on their own [33]. Furthermore, data from the Kenyan Demographic and Health Survey (2008-9) [18] showed 73% of women reported that they either made their own decision, or a joint decision with regard to health care.

It is clear that most of the participants saw their role as separate to their wife’s i.e. they were not a ‘couple’ going through the pregnancy together. Joint couple decision making has been shown to improve the use of facility-based ANC and delivery care and use of skilled birth attendance [34,35]. Efforts focused on couples have been shown to have a greater net impact on the utilisation of facility-based ANC and postnatal care [36]. Our findings would suggest that whilst directing efforts towards couples would be the ideal, until the cultural norm facilitates this, efforts might be misdirected. This might be particularly difficult to overcome in a society where polygamy is the norm. When a man has more than one wife, being part of a ‘couple’ could have a different meaning. Our focus groups comprised both polygamous and non-polygamous participants, which meant we were unable to explore this issue, or indeed to look whether overall perceptions were different between the two. A recent study reported that sociololegal status of a union was an important predictor of communication between couples, with those in polygamous unions having lower communication [37]. However, Nkuoh et al found a surprisingly higher proportion of men in polygamous relationships accompanied their wife to clinic in comparison with men in monogamous relationships [38]. The influence of marital status would thus seem to be an area worthy of further research.

The male role as provider acted as a barrier to accompanying wives to ANC care. Men reported they were too busy for such tasks, particularly as long queues meant delays in being seen. Similar findings have been reported in other African studies [2,39]. The suggestion to overcome this by prioritising couples amplified the theme of male dominance apparent throughout our discussions. It is also seen as a form of discrimination against women attending clinic alone by subjecting them to longer waiting hours [16]. Possibly men could be encouraged to attend by opening clinics on Saturdays when some men are not in paid work – however, many of the local population are subsistence farmers which may limit attendance.

### The role of healthcare provider in men’s involvement during ANC and delivery

Another barrier stemmed from the reported negative attitudes of HCWs to men’s participation in maternity care, which appeared to reinforce marginalisation of men and the notion that pregnancy care is a female domain. Negative staff attitude as an obstacle to the utilisation of facility-based ANC and delivery care has been described in other settings [40,41]. Some participants reported a reluctance to accompany their wives for fear of being ignored or chastised by HCWs. Other studies have reported that HCWs do not allow husbands in with their wives due to congestion and lack of privacy in ANC clinics and labour rooms therefore negating any efforts directed towards encouraging the participation of men in care. [2,42,43]. It is paramount, therefore, that HCW are trained to improve interpersonal communication so as to increase client satisfaction. If our findings are confirmed, future designs for the construction of maternal and child care units should be more couple-friendly allowing for accommodation to include men as well as women, with perhaps separate waiting areas for men or couples. However, as we found that the few men, who were allowed in, chose not to enter the delivery suite, perhaps a starting point would be to have waiting rooms close by so that men are on hand to make decisions and be part of the experience, without having to compromise their beliefs. Kululanga et al, report that informed and motivated male partners in Malawi can be birth companions as long as there is an enabling environment with a positive HCW attitude towards men [15]. On the contrary, Oboro et al, reports that Nigerian women were uncomfortable having their husbands as labour companions due to fear of loss of sexual effectiveness, personal embarrassment and lack of privacy [44]. Consequently, women’s views should be taken into account as this may differ with traditional cultures and believes.

### Other factors affecting health seeking behaviour during pregnancy and delivery

The apparent positive attitude towards facility-based care, along with perceived benefits to accompanying wives, demonstrates that there is potential for change. Ultimately though, despite the positive view – simple economics or logistics might be the driving force determining whether the woman attends hospital for delivery or seeks help from the TBA. The study was conducted in an area where economic activity is focused around subsistence farming or fishing, with scarce resources. Lack of transport and distance to clinic are likely to impede the ability to choose, factors which are widely recognised throughout developing countries [33,42,43]as well as reported by women in the recent Kenya Demographic and Health Survey [18].

### Study limitations

Some limitations of this study need to be considered. Our participants were drawn from a wide age range stratified by area, including men who had lived and worked outside the study area, therefore our findings should be generalised to the parent population. However, they may not be generalizable to other settings with significant differences in health system management, cultural practices regarding pregnancy and delivery care and socio-economic status. During translation and transcription, some data may have been lost or original meanings distorted, but the use of the moderator to proof read the transcripts minimised such loss.

Importantly, it is likely that our findings were skewed by our data collection method. Using a series of male FGDs, with both moderator and note taker being male and of the same cultural background, may well have introduced an element of ‘machismo’. It is believed that gender roles could have been exaggerated within such a group atmosphere. On the other hand, it may also have facilitated a conductive atmosphere in which participants felt comfortable in voicing their true thoughts. We cannot be certain which, if any, was the stronger influence. It would be useful therefore, for future research to explore the views of women on the role of their husbands during pregnancy and delivery, and for men to be interviewed on a one to one basis where they may be less prone to ‘machismo’ but possibly more constrained by the interviewer effect. It would also be important to find out and compare the views of monogamous versus polygamous men, as well as men who accompany their wives for ANC and delivery versus those who do not.

## Conclusion

Findings from our study are likely to have been influenced by the local context and data collection methods. That is, there is a high prevalence of HIV in ANC attendees, and the study was conducted in a polygamous society, using male only FGDs. In this context, despite low participation in accompanying wives to maternity services, many men expressed a positive attitude towards facility-based care for pregnant women, and could proffer many reasons as to why they should accompany their wives. Overall, many men perceived themselves to be the final decision makers on the choice of care provider, occasionally forcing the wife to comply. Although this provides a potential barrier to genuine joint couple decision making, it also offers opportunity and highlights the importance to encourage and promote male involvement in ANC and delivery care. Policies and training of HCW on customer care and better interpersonal communication skills are important to encourage men’s involvement in ANC and delivery care in this patriarchal society. Despite low costs associated with facility-based care, inflexibility over payment methods calls for a workable health insurance cover for all with flexible means for payment of premium”. Lack of transport (and possible wife preference) means that the TBA is still in demand for her services. However, there are practical ways to address these issues, and with attitudes seemingly positive towards involvement of men, it is possible that a shift towards greater male participation could occur in the not too distant future.

## Abbreviations

ANC: Antenatal care; CDC: Centers for Diseases Control and prevention; FGD: Focus group discussion; HCW: Health care workers; HDSS: Health Demographic Surveillance System; KEMRI: Kenya Medical Research Institute; MDG: Millennium Development Goal; MMR: Maternal mortality ratio; PMTCT: Prevention of Mother to Child Transmission (of HIV); VRs: Village reporters.

## Competing interests

The author(s) declare that they have no competing interests.

## Authors’ contributions

TKK was involved in the conception and design of the study, protocol writing, data collection, analysis and interpretation, and in drafting the manuscript. SD was involved in the design of the study, data collection and critical review of the manuscript. MD was involved in the study conception, data collection and critical review of the manuscript. CAA assisted in protocol writing and critically reviewed the manuscript for substantial intellectual input. BP assisted in study design, analysis and interpretation of data and critically reviewed the manuscript. FA assisted in study design and data collection. LM was involved in data analysis and interpretation and critically reviewed the manuscript. KL assisted with field work and critically reviewed the manuscript for substantial intellectual input. FTK was involved in conception and design of the study, protocol writing, drafting and reviewing the manuscript for significant intellectual input. All authors read and approved the final manuscript.

## Pre-publication history

The pre-publication history for this paper can be accessed here:

http://www.biomedcentral.com/1471-2393/13/134/prepub
